# Pancréatite aiguë sévère sur grossesse chez le noir Africain: à propos d’un cas

**DOI:** 10.11604/pamj.2017.26.175.11652

**Published:** 2017-03-27

**Authors:** Ismaïl Lawani, Aboudou Raimi Kpossou, Bruno Noukpozounkou, Freddy Houehanou Rodrigue Gnangnon, Yacoubou Imorou Souaibou, Dansou Gaspard Gbessi, Benjamin Hounkpatin, Fancis Moïse Dossou, Jean-Léon Olory-Togbe

**Affiliations:** 1 Clinique Universitaire de Chirurgie Générale du CHUDOP, Porto-Novo, Bénin; 2 Clinique Universitaire de Gastroentérologie, CNHU-HKM, Cotonou, Bénin; 3 Clinique Universitaire de Chirurgie Pédiatrique CNHU-HKM, Cotonou, Bénin; 4 Clinique Universitaire de Chirurgie Viscérale “A” CNHU-HKM, Cotonou, Bénin; 5 CHU de la Mère et de l’Enfant Lagume, Cotonou, Bénin

**Keywords:** Pancréatite aiguë, grossesse, Acute pancreatitis, pregnancy

## Abstract

La pancréatite aigue est un évènement rare pendant la grossesse. Elle est associée à une forte mortalité maternelle et fœtale. La lithiase biliaire est l’étiologie la plus fréquente, mais dans beaucoup de cas, la cause reste indéterminée. Nous rapportons ici le cas d’une patiente de 37 ans qui a présenté à 29 semaines d’aménorrhée une pancréatite aiguë révélée par une occlusion intestinale aiguë fébrile. Le diagnostic a été fait en per opératoire. Dans les suites opératoires la patiente a fait une fausse couche, puis est décédée au 8^ème^ jour post opératoire.

## Abstract (en)

Acute pancreatitis is rare during pregnancy. It is associated with high maternofoetal mortality. It is mainly caused by biliary lithiasis but, in many cases, the cause of acute pancreatitis is undetermined. We here report the case of a 37-year old patient presenting with acute pancreatitis revealed by acute febrile bowel obstruction at 29 weeks of amenorrhea. The diagnosis was made during surgery. The patient had a miscarriage in the postoperative period. She died on the 8th postoperative day.

## Introduction

La pancréatite aigüe est une pathologie rare au cours de la grossesse. Les calculs biliaires constituent l’étiologie la plus fréquemment rencontrée [1, 2]. Elle survient dans la plupart des cas au 3^ème^ trimestre de la grossesse [2, 3]. Son évolution peut mettre en jeu le pronostic vital maternel et fœtal. L’association pancréatite et grossesse est peu décrite chez le noir africain. Nous rapportons ici un cas de pancréatite aiguë sur grossesse, qui nous permet de faire une revue de littérature sur cette association rare.

## Patient et observation

**Figure 1:**
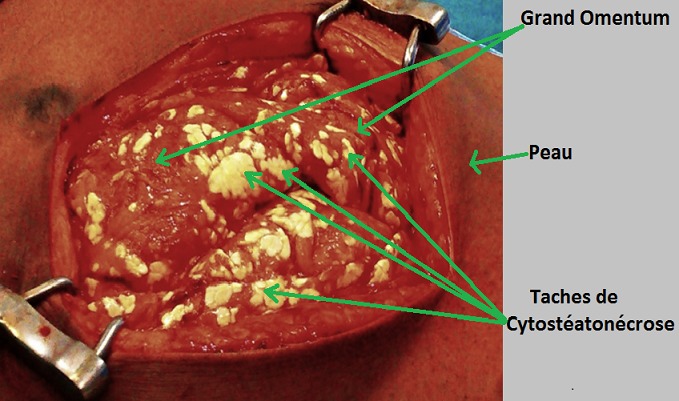
Vue per opératoire de la cytostéaotonécose du grand omentum

**Figure 2:**
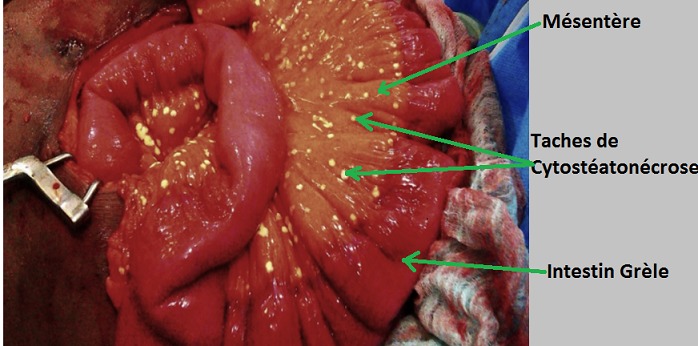
Vue per opératoire de la cytostéatonécrose du mésentère

**Figure 3:**
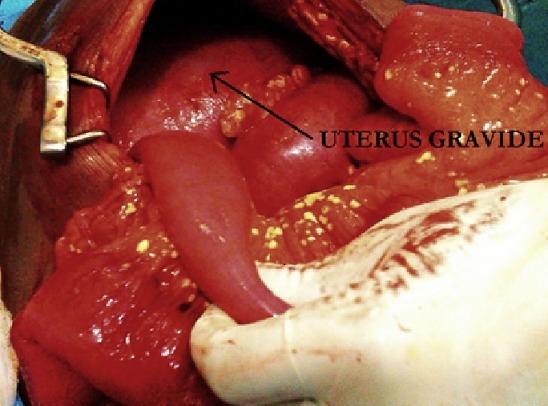
Vue per opératoire de l’utérus gravide avec des taches de cytostéatonécrose

Il s’agissait d’une patiente âgée de 37 ans, de race noire, multipare, sans antécédent particulier, qui a consulté en urgence pour des douleurs aiguës diffuses de l’abdomen, sur une grossesse de 29 semaines d’aménorrhée. L’interrogatoire a retrouvé un début 07 jours avant admission par des douleurs abdominales diffuses avec arrêt des matières et des gaz, et des vomissements alimentaires, puis bilieux. L’examen physique retrouvait : un état général altéré, une conscience conservée, une tension artérielle à 120/80 mm de mercure, un syndrome infectieux (hyperthermie à 38,2°C, tachysphygmie à 120 pulsations par minute), un abdomen douloureux dans son ensemble et météorisé. Le signe de Murphy était absent. Le rythme cardiaque fœtal était normal à 140 battements par minute. La radiographie de l’abdomen sans préparation n’a pas été réalisée à cause de la grossesse; l’échographie abdominale et l’IRM n’étaient pas disponibles en urgence dans notre hôpital. Le bilan biologique montrait: une hyperleucocytose à 19,2 106 /L avec une polynucléose neutrophile à 17,66 106/L, une insuffisance rénale (clearance de la créatinine à 57,5 ml/min), une hypernatrémie à 146 meq/L et une hypokaliémie à 2,8 meq/L. Le taux de Prothrombine était normal à 72%. Les enzymes pancréatiques n’avaient pas été dosées en pré-opératoire. Le diagnostic d’occlusion intestinale aigüe fébrile sur grossesse a été retenu et l’indication d’une laparotomie exploratrice posée. A la laparotomie, on a découvert sur le grand omentum (Figure 1), le mésentère (Figure 2) et le mésocolon transverse des tâches de cytostéatonécrose évocatrices de la pancréatite aiguë nécrotique. La graisse du mésocolon transverse et du ligament gastrocolique étaient infiltrées. La vésicule biliaire était alithiasique et les voies biliaires fines. L’utérus était globuleux (Figure 3) et portait des taches de cytostéatonécrose. On a évoqué une pancréatite aiguë nécrotique. Un bilan biologique prélevé en post opératoire à révélé une hyper amylasémie à 264 UI/l (3.1 fois la limite supérieure de la normale), une hypernatrémie à 146 meq/L, une hypokaliémie à 2,8 meq/l. La lipasémie n’avait pas été dosée car non disponible dans notre hôpital. La prise en charge a associé le repos digestif, la correction des troubles hydro-électrolytiques, les antalgiques, l’antibiothérapie (ceftriaxone + métronidazole) et la tocolyse. L’évolution était marquée au 4^ème^ jour post-opératoire par l’altération de la conscience avec un score de Glasgow à 11/15 (E4V2M5) et l’aggravation des troubles ioniques; la lipasémie alors dosée dans un laboratoire en ville était normale à 32 UI/L. Au 6^ème^ jour post opératoire, elle expulsa son fœtus vivant dont le décès était survenu 6 heures après. Au 8^ème^ jour post opératoire, elle est décédée dans un contexte de choc septique et de troubles hydro-électrolytiques.

## Discussion

La pancréatite aigüe survient rarement au cours de la grossesse. La fréquence rapportée varie de 1 pour 1000 à 3 pour 10000 naissances [1]. L’étiologie la plus fréquemment rencontrée est biliaire à 70 % [1, 2]. On retrouve aussi l’hyper-triglycéridemie, et d’autres étiologies moins communes comme l’hyperpara-thyroidie, l’auto-immunité et l’alcool. Les pancréatites aigues sur grossesse drogues-induites sont rares. Les diurétiques thiazidiques [2] et la metformine [4] ont été incriminés. Des cas de pancréatite aigüe idiopathique ont été également rapportés au cours de la grossesse [5]. Des facteurs de risque ont été retrouvés dans la survenue de la pancréatite aiguë pendant la grossesse. Parmi ces facteurs de risque identifiés [2], la multiparité est le principal facteur de risque présenté par notre patiente. D’après Pitchumoni [1], plus de 50% des pancréatites aigües surviennent au 3ème trimestre de la grossesse, comme chez notre patiente. La physiopathologie est complexe. En effet, au cours du deuxième et du troisième trimestre de la grossesse, on note une sursaturation de la bile en rapport avec l’augmentation de l’excrétion du cholestérol dans la bile par rapport aux acides biliaires et aux phospholipides. La stagnation de cette bile sursaturée dans la vésicule biliaire induit la formation de cristaux du cholestérol et finalement de calculs biliaires [1]. Par ailleurs, au troisième trimestre, l’utérus gravide comprime le conduit cholédoque et peut entraîner une pancréatite aigüe [6]. La pancréatite aigüe pendant la grossesse peut être associée avec un HELLP Syndrome ou une pré éclampsie qui augmentent la mortalité fœtale [6]. L’échographie abdominale est l’examen d’imagerie idéal pour diagnostiquer la pancréatite aiguë au cours de la grossesse parce qu´il n´a aucun risque d’irradiation et est utile pour détecter les conduits pancréatiques dilatés et les pseudo kystes [7]. Cependant, sa réalisation est difficile parce qu´un utérus agrandi rend invisible le pancréas. La cholangio-pancréatographie par résonnance aimantée est une alternative avec une sensibilité de 90% sans exposer ni la mère ni le fœtus aux radiations ionisantes. Ces examens complémentaires n’ont pas été réalisés chez notre patiente.

Le diagnostic de pancréatite aigüe a été retenu chez cette patiente principalement sur les douleurs, la cytostéatonécrose et l’amylasémie. Mais la lipasémie est le marqueur biologique le plus spécifique pour le diagnostic de la pancréatite aigüe. Une élévation de la lipasémie supérieure à 3 fois la normale est fortement évocatrice d’une pancréatite aigüe [8]. Au cours de la pancréatite aigüe, elle apparaît précocément, atteint son maximum en 48 heures et décroit pour disparaitre au bout d’une dizaine de jour. Cette cinétique explique la normalisation de la lipasémie dosée chez notre patiente douze jours après le début de la symptomatologie. On distingue par rapport à la sévérité de la pancréatite aigüe 2 types : la pancréatite aigüe bénigne (sans défaillance d’organes, ni de complications locales ou systémiques) et la pancréatite aigüe grave (avec une défaillance d’organe, une complication systémique ou une complication locale comme la nécrose pancréatique, les collections liquidiennes péri pancréatiques, le pseudo kyste) [9]. La prise en charge des pancréatites aigues prend en compte l’hydratation, l’oxygénothérapie, le traitement antalgique et la diète pour mettre au repos la fonction endocrine du pancréas, prévenant ainsi de l’autodigestion pancréatique [1]. Cependant, dans la pancréatite aigüe grave, le traitement doit inclure la nutrition entérale ou à défaut parentérale. La nutrition parentérale totale expose au risque d´infections et désordre métabolique. La nutrition entérale est physiologique et aide à maintenir la flore intestinale [7]. En dehors d’une angiocholite associée, l’usage d´antibiotiques dans la pancréatite aiguë pour protège contre l’infection de la nécrose est controversé [2, 3]. Lorsqu’on les utilise, le choix des antibiotiques est difficile, à cause du risque tératogène. L’amoxicilline et le métronidazole utilisé chez notre patiente, mais aussi le sulbactam, la pipéracilline et le tazobactam sont utilisables sans risque [2]. Dans les pancréatites aigues bénignes, le pronostic est excellents tant pour la mère que pour le fœtus. Pour la pancréatite aigüe grave, le pronostic maternel est réservé. Notre patiente a accusé d’un retard de 7 jours avant sa prise en charge. Ce retard dans la prise en charge aggrave le pronostic des patientes selon certains auteurs. Ainsi, Gangat [10] au Pakistan a trouvé 30.76% de mortalité maternelle et reliait ce constat au retard diagnostic et thérapeutique.

## Conclusion

L’association pancréatite aigüe sévère et grossesse est rare. Ses étiologies sont nombreuses et dominées par les calculs biliaires et l’hyper-triglycéridemie. Le diagnostic positif est basé sur le dosage de l’amylasémie et/ou la lipasémie. Chez notre patiente aucune étiologie n’a été retrouvée. Le retard diagnostic et thérapeutique a été fatal au couple mère enfant.
